# Using default constraints of the spindle assembly checkpoint to estimate the associated chemical rates

**DOI:** 10.1186/2046-1682-5-1

**Published:** 2012-01-19

**Authors:** Khanh Dao Duc, David Holcman

**Affiliations:** 1 Institute for Biology (IBENS), Group of Computational Biology and Applied Mathematics, Ecole Normale Supérieure, 46 rue d'Ulm 75005 Paris, France

## Abstract

**Background**

Default activation of the spindle assembly checkpoint provides severe constraints on the underlying biochemical activation rates: on one hand, the cell cannot divide before all chromosomes are aligned, but on the other hand, when they are ready, the separation is quite fast, lasting a few minutes. Our purpose is to use these opposed constraints to estimate the associated chemical rates.

**Results**

To analyze the above constraints, we develop a markovian model to describe the dynamics of Cdc20 molecules. We compute the probability for no APC/C activation before time t, the distribution of Cdc20 at equilibrium and the mean time to complete APC/C activation after all chromosomes are attached.

**Conclusions**

By studying Cdc20 inhibition and the activation time, we obtain a range for the main chemical reaction rates regulating the spindle assembly checkpoint and transition to anaphase.

## I. Background

A fundamental step in cell division consists in the alignment of each pair of chromosomes. This process occurs during metaphase, where centrosome nucleated microtubules interact with the chromosomes kinetochores to build the mitotic spindle. Only after all chromosomes have become aligned at the metaphase plate and when every kinetochore is properly attached to a bundle of microtubules, the cell enters anaphase. To prevent premature progression to anaphase, even if all-but-one of the kinetochores have been attached and the chromosomes are aligned, unattached or improperly attached kinetochores generate a signal inhibiting the anaphase activators. This process is called the spindle assembly checkpoint (SAC).

Although the exact mechanisms of the SAC and anaphase processes are still unclear, several key steps have been identified. Sister chromatids are initially bound by proteins such as cohesin. During anaphase onset, separase protein cleaves cohesin, thus allowing the sister chromatids to separate [1]. Usually, separase is prevented from cleaving cohesin through its association with another protein called securin. Securin can be ubiqitylated by the activated Cdc20-anaphase promoting complex/cyclosome (Cdc20-APC/C) at the kinetochores [2]. However, when the kinetochores are not all properly attached, the SAC enables the ubiquitylation and inhibition of Cdc20 binding with APC/C [2,3]. The mechanisms leading to Cdc20 ubiquitylation involve several proteins such as Mad2, BubR1, Bub3 [4]. Current models [5-10] of the checkpoint propose that Mad2 protein has a crucial role either to sequester Cdc20, or acts in conjunction with the BubR1 and Bub3 proteins to form an inhibitor called the Mitotic Checkpoint Complex (MCC). Mad2 changes conformation to bind Cdc20 tightly via a safety belt mechanism [5,7,8], while another inhibitory complex comprised of BubR1 and Bub3 has also been identified [9,10].

The SAC has been modeled at a molecular level, however the parameters used [11-14] may not necessarily reflect *in vivo *dynamics [15]. For example, these modelings do not take into account the finite number of binding sites for Cdc20. In addition, the constant flux assumption [11] made for molecules reaching a kinetochore impacts the APC/C activation, leading to an overestima-tion for the catalytic activity. We shall revisit here some of these major assumptions in the construction of our model. An improvement of these models was recently achieved [16,17] by taking into account the finite number of binding sites at kinetochores [16], leading also to an estimate for the MCC chemical rates associated with SAC.

In the present article, our purpose is to study the inhibition followed by its fast activation of Cdc20, which is the key activator of the anaphase promoting complex. As the number of kinetochores implied in the SAC is small, the forward binding rate of a chemical reaction as it is classically computed in the continuously concentrated limit cannot be applied. To adequately describe chemical reactions in microdomains [18,19], where targets such as kinetochores have to be reached by the anaphase activators, we use a stochastic approach. Using Markovian equations [18,20] to account for the binding dynamic associated with a finite number of molecules, we compute the time dependent probability that the spindle is not initiated before time *t *(formula 23) and then the mean time to induce anaphase (implicit formula 29). We apply our analysis to PTK2 cells and thus, we obtain some quantitative constraints on the Cdc20 production rate and the MCC concentration to guarantee strong inhibition of Cdc20 by the SAC. Using different parameter values (cell size, number of chromosomes...), our method can be extended to other cell types and organisms, providing a general framework to study the dynamics of activators during the spindle checkpoint and the anaphase transition.

## II. Methods

### Markovian modeling of APC/C activation and Cdc20 inhibition

**Figure 1:**
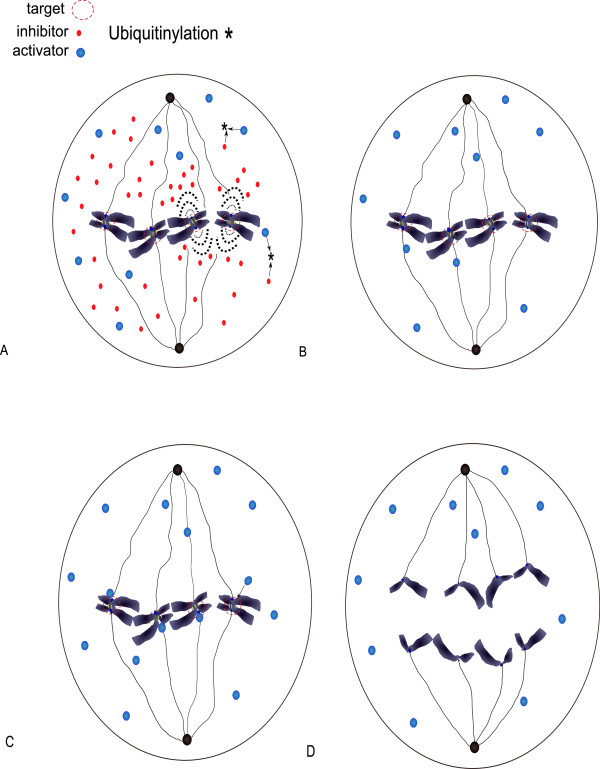
**A schematic view of the spindle assembly checkpoint and anaphase**. **A**: Before all chromosomes are all attached, the mitotic checkpoint complex inhibits the Cdc20 molecules binding with APC/C to prevent premature separation of sister chromatids. This signal ubiquitylates Cdc20. **B**: When all chromosomes are properly attached, the inhibiting signal is shut down. **C **and **D**: Activation of APC/C triggers the separation of the chromatids and ultimately the anaphase.

**Figure 2:**
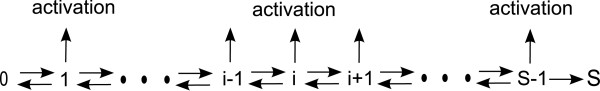
**Markov diagram for the probability of number of Cdc20 molecules**. Cdc20 at state *k *is generated and destroyed at rate λ(*S -- k*) and *k*_-1_*k *respectively. Diffusing Cdc20 molecules bind to the APC/C complex to trigger the separation of sister chromatids.

We describe here the time evolution of the joint probability distribution of Cdc20 molecules and of APC/C complex activation, the later being responsible for the chromosome separation. In this model, APC/C is located on the chromosomes (figure 1), and is a target of the Cdc20 molecule, although there are some conflicting evidences that APC/C is located on the kinetochores [21]. This assumption can affect the binding rate, but does not impact the construction of our model. For a cell containing *N *chromosomes, the targets of the Cdc20 molecules are the *N *associated kinetochores, containing the APC/C complexes. When a Cdc20 molecule reaches a kinetochore, it activates the APC/C complex and this can trigger a cascade of reactions (detailed in the background section) leading to the separation of the sister chromatids. The goal of the spindle assembly checkpoint signal is to prevent this activation of APC/C by Cdc20, when at least one of the chromosomes is not properly attached to the microtubules responsible for the chromatids migration. The spindle assembly checkpoint signal consists in the production of proteins such as Mad2, BubR1 and Mad3 [4], generated by unattached kinetochores. These proteins diffuse in large quantity in the cell to inhibit the APC/C binding by Cdc20 molecules. Indeed, these proteins form with Cdc20 a complex called mitotic checkpoint complex (MCC). Similar complexes can be found in yeast, in which a BubR1-related Mad3 protein might inhibit Cdc20 as a pseudo-substrate [22-24]. The formation of this complex results in Cdc20 ubiquitylation, which prevents APC/C activation. In our model, MCC will represent the complex of inhibitory proteins before it binds Cdc20, in contrast with the usual terminology where MCC includes Cdc20. Cdc20 molecules are produced from the dissociation of a complex [25], which could be a subcomplex of Mad2 and Cdc20 resulting from MCC:Cdc20 disassembly, promoted by p31 [26]. In summary, the above chemical reactions can be summarized as

(1) $$\begin{array}{c} Complex:Cdc20\overset{\lambda }{\to }Complex+Cdc20\;\left(\text{production}\right)\\ Cdc20+MCC\overset{k_{-1}}{\to }MCC:Cdc20\;\left(\text{ubiquitylation}\right)\\ Cdc20+APC/C\overset{\mu }{\to }APC/C:Cdc20\;\left(\text{activation}\right). \end{array}$$

where the rate λ measures the production of Cdc20 and *k*_-1 _the degradation, while *μ *is the arrival rate for a CdC20 to an APC/C site. We shall compute in the next paragraphs the joint probability

(2) $$p_{k}\left(t\right)=Pr\left(|Cdc20|\left(t\right)=k,\text{no}\;\text{activation}\;\text{occurred}\;\text{before}\;\text{time}\;t\right)$$

that the APC/C is not activated by any free Cdc20 molecule at time *t*. To compute this probability, we first derive a Markov equation. The difficulty is that this joint probability contains a discrete variable counting the number of Cdc20 molecules and a binary one, which monitors whether or not an activation of APC/C by Cdc20 has occurred before time *t*. The state space of this Markov process is completed by adding a state describing that activation occurred before time t. It is modeled as an absorbing state of probability

(3) $$p^{*}\left(t\right)=Pr\left(\text{activation}\;\text{occurred}\;\text{before}\;\text{time}\;t\right),$$

which accounts for all the activations which have happened before time *t *from all the states *k*. Starting with k active Cdc20 molecules and no activation, there are three possible transitions (figure 2): 1) one Cdc20 molecule is inhibited, so that k-1 active molecules are left 2) one Cdc20 molecule activates the APC/C 3) one Cdc20 molecule is generated, leading to the transition from k to k+1 active molecules. Thus, the probabilities *pk *satisfy the chemical master equations [18,20]

(4) $$\begin{array}{ll} {ṗ}_{0} & =-\lambda Sp_{0}+k_{-1}p_{1}\;\\ {ṗ}_{k} & =-\left(\lambda \left(S-k\right)+\left(\mu N+k_{-1}\right)k\right)p_{k}\;\\ & \;+\lambda \left(S-k+1\right)p_{k-1}+k_{-1}\left(k+1\right)p_{k+1},\;\text{for}\;1\leq k\;\\ {ṗ}^{*} & =\underset{k}{\sum }\mu Nkp_{k}.\;\\ & \; \end{array}$$

The production rate of Cdc20 molecules is proportional to the number of remaining available complex molecules given by λ(*S *- *k*). Indeed the Cdc20 molecules are produced during metaphase by dissociation from a pool of complexes, which limits the level of Cdc20 to a maximum of *S *molecules [25]. In addition, we shall emphasize that there is another interpretation of equation (4): indeed, dissociation of the MCC:Cdc20 complex, producing Cdc20 molecule, leads also to equation (4). When none of the *N *target kinetochores have been activated, the arrival rate for a Cdc20 molecule to an APC/C is *μN*, where $\mu =\frac{1}{\tau }$ and *τ *is the mean time for a Cdc20 molecule to reach the APC/C site. This mean time can be approximated by [27-29]

(5) $$\mu =\frac{3rD}{\pi R^{3}},$$

where *D *is the diffusion coefficient of a Cdc20 molecule, *a *is the radius of the APC/C complex, *R *the radius of the cell. As the mechanisms underlying the production and the regulation of MCC are still unclear, we consider that the MCC concentration is homogeneous and remains constant over time to guarantee a robust inhibition of Cdc20. Thus, k_-1 _is given by the Smoluchovski formula for the binding rate of a Brownian particle

(6) $$k_{-1}=2\pi bD\left[MCC\right],$$

where [*MCC*] is the concentration of MCC, uniform over the cell and *b *is the radius of the Cdc20 binding site. When the SAC starts, no free Cdc20 molecules are present in the cell, thus we choose for the initial conditions *pk*(*0*) = *δ*_*k*,0_. Because there can only be *S *Cdc20 molecules, we have for all time t, *pk*(*t*) = 0 and *k *>*S.*

### The probability for no activation

To quantify the inhibition capacity of the SAC, we estimate the probability *P*(*t*) that at time *t*, no APC/C has been activated, so that no chromosomal migration could have been initiated. This probability is given by

(7) $$P\left(t\right)=\overset{+\infty }{\underset{k=0}{\sum }}p_{k}\left(t\right).$$

We shall compute *P*(*t*) using the generating function

(8) $$f\left(t,x\right)=\overset{+\infty }{\underset{k=0}{\sum }}p_{k}\left(t\right)x^{k}.$$

Using equation (4), *f *satisfies a first order PDE

(9) $$\frac{\partial f}{\partial t}=\lambda S\left(x-1\right)f+\left(-\lambda x^{2}+\left(\lambda -\mu N-k_{-1}\right)x+k_{-1}\right)\times \frac{\partial f}{\partial x}.$$

Using the characteristics method, we look for a solution of

(10) $$Ẋ=\lambda X^{2}-\left(\lambda -\mu N-k_{-1}\right)X-k_{-1},$$

which is of Riccati type. From the classical substitution $x=-\frac{1}{\lambda }\frac{u^{\prime }}{u}$ we obtain the linear second order differential equation

(11) $$u^{\prime \prime }+\left(\lambda -\mu N-k_{-1}\right)u^{\prime }-k_{-1}\lambda u=0.$$

Thus, the solution for characteristics is

(12) $$x_{C}\left(t\right)=-\frac{1}{\lambda }\left(\frac{r_{1}e^{r_{1}t}+r_{2}Ce^{r_{2}t}}{e^{r_{1}t}+Ce^{r_{2}t}}\right),$$

where *C *is a constant and *r*_1 _and *r*_2 _are the two roots of the quadratic polynomial associated with (11)

(13) $$r_{1}=\frac{1}{2}\left(-\lambda +\mu N+k_{-1}+\sqrt{{\left(-\lambda +\mu N+k_{-1}\right)}^{2}+4k_{-1}\lambda }\right)$$

(14) $$r_{2}=\frac{1}{2}\left(-\lambda +\mu N+k_{-1}-\sqrt{{\left(-\lambda +\mu N+k_{-1}\right)}^{2}+4k_{-1}\lambda }\right).$$

Along one of these characteristics, *f *satisfies the linear first order ODE

(15) $$\frac{df\left(t,x_{C}\left(t\right)\right)}{dt}=\lambda S\left(x_{C}\left(t\right)-1\right)f\left(t,x_{C}\left(t\right)\right).$$

The general solution for equation (15) is

(16) $$f\left(t,x_{C}\left(t\right),K\right)=K\text{exp}\left(\lambda S\int_{0}^{t}\left(x_{C}\left(u\right)-1\right)du\right).$$

At time *t *= 0, we have *p*_*k*_(0) = *δ*_*k*0_, initial conditions set *K *= 1. To find *p*(*t*), we shall select the characteristic for which at time *t, x*_*C*_(*t*) = 1. Solving this, yields to

(17) $$C\left(t\right)=-e^{\left(r_{1}-r_{2}\right)t}\frac{\lambda +r_{1}}{\lambda +r_{2}},$$

and we obtain the characteristic

(18) $$x_{C\left(t\right)}\left(u\right)=-\frac{1}{\lambda }\left(\frac{r_{1}-r_{2}\left(\frac{\lambda +r_{1}}{\lambda +r_{2}}\right)\text{exp}\left[\left(r_{1}-r_{2}\right)\left(t-u\right)\right]}{1-\left(\frac{\lambda +r_{1}}{\lambda +r_{2}}\right)\text{exp}\left[\left(r_{1}-r_{2}\right)\left(t-u\right)\right]}\right).$$

Finally, the probability *P*(*t*) of no activation is

(19) $$\begin{array}{c} P\left(t\right)=\text{exp}\left[-\lambda S\left(t+\frac{1}{\lambda }\right)\ldots \right)\\ \left(\left(\;\;\;\;\times \int_{0}^{t}\frac{r_{1}-r_{2}\left(\frac{\lambda +r_{1}}{\lambda +r_{2}}\right)e^{\left(r_{1}-r_{2}\right)u}}{1-\left(\frac{\lambda +r_{1}}{\lambda +r_{2}}\right)e^{\left(r_{1}-r_{2}\right)u}}du\right)\right] \end{array}$$

(20) $$\begin{array}{c} =\text{exp}\left[-\lambda S\left(t+\frac{1}{\lambda }\left[r_{1}u-\text{ln}\left(1-\left(\frac{\lambda +r_{1}}{\lambda +r_{2}}\right)\right)\right)\right)\right)\\ \;\;\;\left(\left({\left(\left(\times e^{\left(r_{1}-r_{2}\right)u}\right)\right]}_{0}^{t}\right)\right] \end{array}$$

(21) $$=e^{-\lambda St-r_{1}St}{\left(\frac{-\lambda -r_{2}+\left(\lambda +r_{1}\right)e^{\left(r_{1}-r_{2}\right)t}}{\left(r_{1}-r_{2}\right)}\right)}^{S}$$

(22) $$=e^{-\lambda tS}{\left(\frac{\left(\lambda +r_{1}\right)e^{-r_{2}t}-\left(\lambda +r_{2}\right)e^{-r_{1}t}}{\lambda \left(r_{1}-r_{2}\right)}\right)}^{S}$$

Finally,

(23) $$P\left(t\right)={\left(\frac{\left(\lambda +r_{1}\right)e^{-\left(\lambda +r_{2}\right)t}-\left(\lambda +r_{2}\right)e^{-\left(\lambda +r_{1}\right)t}}{r_{1}-r_{2}}\right)}^{S}$$

**Figure 3:**
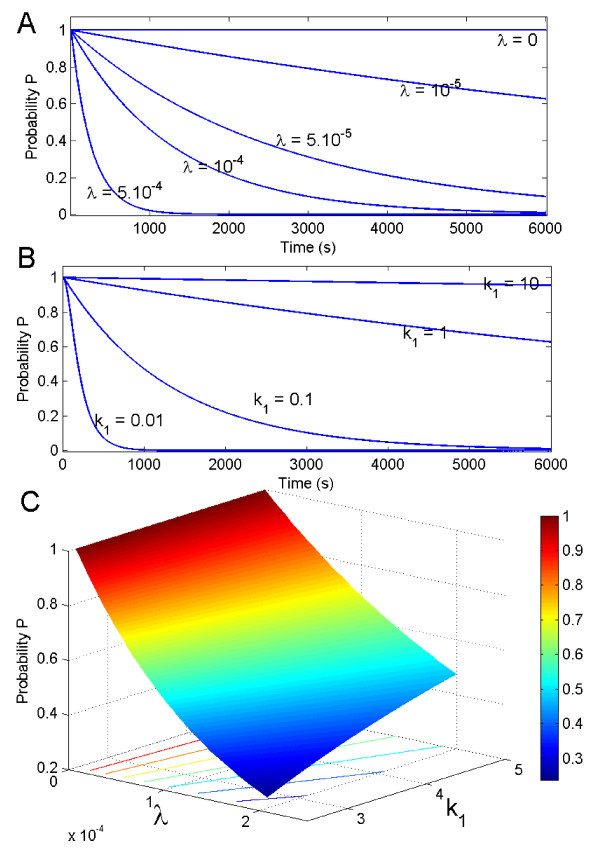
**The probability *P *for no activation during the SAC is represented as a function of the time and the rates λ and *k*_-1_**. **A **:We plot *P*(*t*) as a function of time for different values of λ and *k*_-1 _= 1. **B**: as a function of time for different values of k_-1 _and λ = 0.1. **C**: at time t = 1200s as a function of λ and *k*_-1_. The parameters are given in table 1.

*P *is a decreasing function of time, and remains constant for λ = 0 and *μ *= 0. In figure 3, we plot *P *as a function of time for different values of λ and k_-1_, and as a function of λ and *k*_-1 _at a given time. It is a decreasing function of λ (increasing the Cdc20 production rate decreases the probability of activation) and a decreasing function of k_-1 _(increasing the inhibition of Cdc20 increases the probability for no activation).

### The distribution of Cdc20 at equilibrium

After the last chromosome attached and thus all kinetochores are properly positioned, the inhibition of APC/C:Cdc20 binding is suppressed and anaphase can start. The initial condition for the number of Cdc20 molecules for this new phase is the one obtained at equilibrium from the previous phase, in which Cdc20 is produced and destroyed by the SAC. When there are *k *Cdc20 molecules, the production rate is given by λ(*S*-*k*) and the destruction rate *k*_-1_(*k*+1). Thus, the probability *p*_*k*_(*t*|*NA*) that k Cdc20 molecules are inside the cell, conditioned that no activation has occurred, satisfies the Master equations

(24) $$\begin{array}{c} {ṗ}_{0}\left(t|NA\right)=-\lambda Sp_{0}\left(t|NA\right)+k_{-1}p_{1}\left(t|NA\right)\\ {ṗ}_{k}\left(t|NA\right)=-\left(\lambda \left(S-k\right)+k_{-1}k\right)p_{k}\left(t|NA\right)\\ \;\;\;\;\;\;+\lambda \left(S-k+1\right)p_{k-1}\left(t|NA\right)\\ \;\;\;\;\;\;+k_{-1}\left(k+1\right)p_{k+1}\left(t|NA\right). \end{array}$$

The equilibrium probabilities *p*_*k*_(∞) and the mean number $\bar{N}$ for such a system is [30]

(25) $$p_{k}\left(\infty \right)=\frac{\left(\begin{array}{c} S\\ k \end{array}\right){\left(\frac{\lambda }{k-1}\right)}^{k}}{{\left(1+\frac{\lambda }{k-1}\right)}^{S}}$$

(26) $$\bar{N}=\frac{S\lambda /k_{-1}}{1+\lambda /k_{-1}}.$$

When the SAC is suppressed, Cdc20 is no longer inhibited and can activate APC/C to trigger anaphase. Using the distribution computed here we compute in the next section the mean time for complete separation of sister chromatids during anaphase.

### Activation of APC/C

When all kinetochores are properly attached, the SAC is shut down and the activation of APC/C:Cdc20 complex triggers a cascade of reactions leading to cohesin ubiquitylation at the chromosome sites [1]. To study the time for such activation, we consider that production and degradation of MCC are fast enough so that the MCC concentration decreases rapidly once all the kinetochores are attached. In that case, we can neglect the transient time for the rate *k*_-1 _to decay to 0 and thus we take the equilibrium Cdc20 concentrations as the initial conditions for the activation of APC/C. For our analysis, we further consider that the time for all kinetochores to be attached is not too short compared to the degradation and production time scale, so that the CdC20 concentration is close to equilibrium. When there are *k *Cdc20 present in the cell and *m *of them are bound to APC/C, it results that the association rate is *μ*(*N *- *m*)(*k *- *m*) [18]. We shall now estimate the joint probabilities that there are *k *Cdc20 molecules, and *m *activated APC/C by Cdc20

$$p_{k,m}(t)=Pr(|CDC20|=k,m\;\text{activated}\;\text{APC}/\text{C}\;\text{by}\;\text{Cdc}20)$$

**Figure 4:**
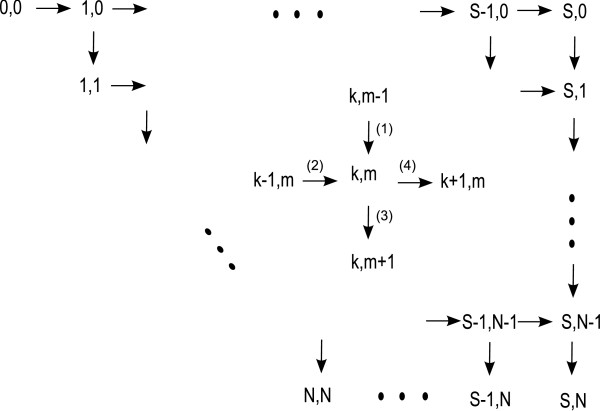
**Schematic representation of the Markov Chain associated with the joint probability *p*_*k*,*m*_(*t*) to have *k *bounds APC/C and *m *free Cdc20 molecules**.

From the state (*k*,*m*), the transition rate to activation of APC/C located on another kinetochore is then *μ*(*k *- *m*)(*N *- *m*). Thus, we get the following Markov chain (represented in figure 4)

(27) $$\begin{array}{ll} {ṗ}_{0,0} & =-\lambda Sp_{0,0}\;\\ {ṗ}_{k,0} & =-\left(\lambda \left(S-k\right)+\mu Nk\right)p_{k,0}+\lambda \left(S-k+1\right)p_{k-1,0}\;\\ {ṗ}_{k,k} & =-\lambda \left(S-k\right)p_{k,k}+\mu \left(N-k+1\right)p_{k,k-1}\;\\ {ṗ}_{k,m} & =-\left(\lambda \left(S-k\right)+\mu \left(N-m\right)\left(k-m\right)\right)p_{k,m}\;\\ & \;+\lambda \left(S-k+1\right)p_{k-1,m}\;\\ & \;\;+\mu \left(N-m+1\right)\left(k-m+1\right)p_{k,m-1},\;\\ & \; \end{array}$$

with the initial condition

(28) $$p_{k,m}\left(0\right)=\frac{\left(\begin{array}{c} S\\ k \end{array}\right){\left(\frac{\lambda }{k-1}\right)}^{k}}{{\left(1+\frac{\lambda }{k-1}\right)}^{S}}\delta_{0,m},$$

computed in equation (25). In that case, the mean time *τ *that all APC/C are activated is obtained by analyzing a continuous markov process that reaches a given threshold [20]. Using formula 12 of [20], the mean time to threshold is expressed as a sum

(29) $$\tau =\overset{N-1}{\underset{k=0}{\sum }}\overset{S}{\underset{m=0}{\sum }}a_{k,m},$$

where $a_{k,m}=\int_{0}^{\infty }p_{k,m}\left(t\right)dt$. Integrating the system of equation (28) from 0 to + ∞ with the initial conditions

(30) $$p_{k,m}\left(0\right)=\delta_{m,0}\frac{\left(\begin{array}{c} S\\ k \end{array}\right){\left(\frac{\lambda }{k_{-1}}\right)}^{k}}{{\left(1+\frac{\lambda }{k_{-1}}\right)}^{S}},$$

leads to

(31) $$\begin{array}{ll} -p_{0,0}\left(0\right) & =-\lambda Sa_{0,0}\;\\ -p_{k,0}\left(0\right) & =-\left(\lambda +\mu N\right)\left(S-k\right)a_{k,0}+\lambda \left(S-k+1\right)a_{k-1,0}\;\\ 0 & =-\lambda \left(S-k\right)a_{k,k}+\mu \left(N-k+1\right)a_{k,k-1}\;\\ 0 & =-\left(\lambda \left(S-k\right)+\mu \left(N-m\right)\left(k-m\right)\right)a_{k,m}\;\\ & \;+\mu \left(N-m+1\right)\left(S-k\right)a_{k,m-1}\;\\ & \;\;+\lambda \left(S-k+1\right)a_{k-1,m}.\;\\ & \; \end{array}$$

**Figure 5:**
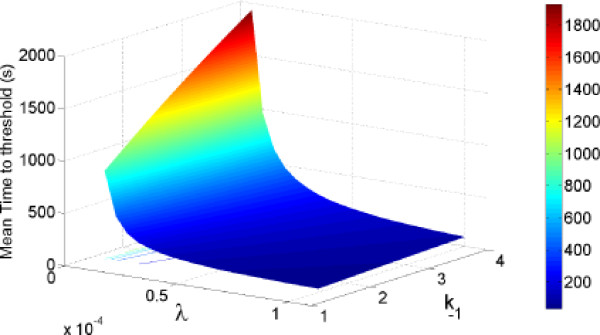
**The time *τ *is plotted as a function of the parameters λ and *k*_-1_**. The parameter valuers are given in table 1.

In practice, we solve this linear system of equations numerically and in figure 5, we plot the mean *τ *as a function of the parameters *k*_-1 _and λ. We find that *τ *is a decreasing function of λ (the faster Cdc20 is produced, the faster the threshold of bindings is reached) and an increasing function of the rate *k*_-1 _(inhibition decreases the number of Cdc20 at equilibrium and thus the time to reach the threshold, after the source of inhibition is terminated). These variations go in the opposite direction compared to the probability of no activation during the SAC. Thus we expect that using the probability *P *and this mean time *τ *will lead to limit the range of the parameters λ and *k*_-1 _as we will describe now.

## III. Results

### Quantitative constraints on the rates λ and *k*_-1_

We now apply our previous modeling to determine the rates of production λ and the backward binding rate *k*_-1_. Indeed, during SAC, a strong inhibition signal imposes that the probability for no activation remains very high and thus, the degradation rate *k*_-1 _has to be high enough compared to the production rate λ. In contrast, a fast activation during anaphase forces the mean time to activate all the kinetochores to be short, thus the production rate λ has to be high. These opposite constraints allow us to determine a range for the parameters λ and *k*_-1_. We use the following quantitative constraints.

1. First, the probability *P *of no activation remains high enough during the time *τ*_1 _where all chro mosomes get properly attached in the metaphase plate. It has been estimated that *τ*_1 _≈ 20 min [31]. Thus by fixing a threshold of 0.95 for the probabil ity *P*(*τ*_1_) that no activation occurred before time *τ*_1_

(32) $$P\left(\tau_{1}>20\text{min}\right)\geq 0.95\;\left(C1\right).$$

2. Second, during the anaphase onset, the time 〈*τ*_*s*_〉 for all chromosomes to get separated is short. Since APC/C activation triggers the chromosome sep aration, we can consider that *τ*_*S *_is the time for all APC/C to get activated. Indeed, biophysical data [32] suggest that *τ*_*s *_should be limited in time *τ' *≈ 10 min. Thus,

(33) $$\left\langle \tau_{S}\right\rangle \leq \tau^{\prime }\;\left(C2\right).$$

**Figure 6:**
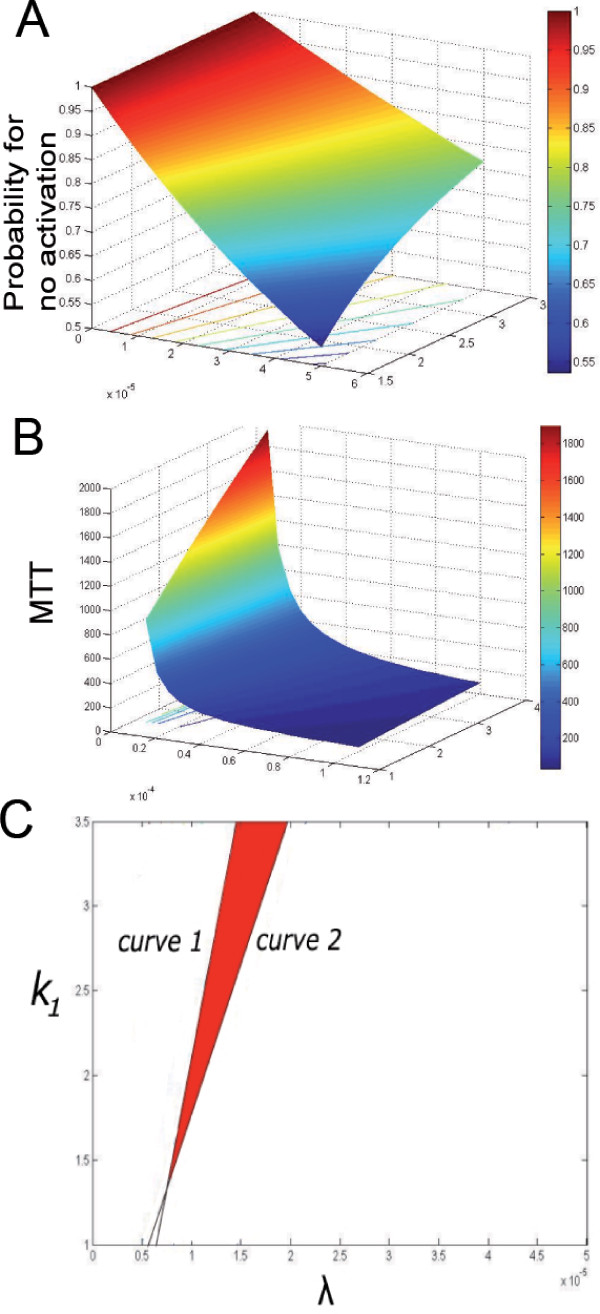
**Representation of the domain Ω (red)**. **A**: The probability for no activation at time *τ *= 10*min *as a function of parameters λ and *k*_-1_. Other parameters are those of table I B : The mean time to threshold as a as a function of parameters λ and *k*_-1_. Other parameters are those of table I. C : The curve 1 is given as the level line associated *τ *= 10 min in figure B. The curve 2 is the level line associated with the probability P given by *P*(*τ*) = 0.95 in figure A. These two curves determine the domain Ω satisfying conditions C1 and C2.

Using formula (23) for the probability *P*(*τ*_1_) and integrating numerically the time 〈*τ*_*S*_〉 from the matrix equation (31), we determine a range of validity for these parameters by a geometrical domain Ω represented in figure 6, as the intersection Ω = Ω_1 _∩ Ω_2_, where

(34) $$\Omega_{1}=\left\{\left(\lambda ,k_{-1}\right)s.t.P\left(\tau_{1}\left(\lambda ,k_{-1}\right)>20\text{min}\right)\geq 0.95\right\}$$

and

(35) $$\Omega_{2}=\left\{\left(\lambda ,k_{-1}\right)s.t.\left\langle \tau_{S}\right\rangle \left(\lambda ,k_{-1}\right)\leq \tau^{\prime }\right\}.$$

We tested the prediction of our model on PTK2 cells, originating from kangaroo rat kidney, used in studies on mitosis because there are only a few large chromosomes and the cells remain flattened during mitosis. For these cells, the concentration of bound complex from which Cdc20 is produced is approximately 50 nM [25] during interphase. This concentration is of the same order as the one reported in [13], and is equivalent to 3000 molecules restricted in a volume of 100 *μm*^3 ^(for a flat cell of size 10 μm and of height 1 *μm*, leading to a volume of 10 × 10 × 1 *μm*^3^). For larger cells, the number of molecules can be multiplied by 10 or 100. Thus, during the SAC, it is tempting to think that the system escapes the stochastic limit. However, because the number of Cdc20 is small at early metaphase and limited by the inhibition of MCC, the stochastic regime is still controlling the behavior of the system and in addition, the inhibition is strong enough to maintain a low level of Cdc20. In figure 6, we represented the two domains Ω_1 _and Ω_2_, the first is on the left of curve 1, while the second is on the right side of curve 2. Our analysis can be generalized by changing the two conditions C1 and C2 for specific cell types. The other parameters are summarized in table 1. Surprisingly, Ω is not bounded, but it provides an interesting and new range for the parameters.

**Table I:** 

Parameter	Description	Value
*V*	cell volume	≈ 100 *μm *^3 ^[25]
*D*	Diffusion coefficient of Cdc20	*D *= 20 *μm*^2^/*sec *[25]
*a*	radius of APC/C complex	≈ 10^-2 ^*μm *[3]
*b*	radius of MCC binding site	≈ 2*nm *[37]
*N*	Number of chromosomes	13 [25]
*S*	Initial number of complexes	3000 [25]
*μ*	binding rate $\left(=\frac{4aD}{V}\right)$	≈ 2.10^-4^*s*^-1^

## IV. Discussion and Conclusion

Based on the two main constraints *C*1 and *C*2, we presented here a Markovian analysis to estimate two fundamental rates regulating the spindle assembly checkpoint. This idea of using physical and timing/inhibition constraints was used before [11] to infer SAC characteristics and compare different models. In a different mathematical framework, our study is based on describing precisely the role of the SAC, which is to prevent a premature separation of chromosomes. Such an event is stochastic, determined by an accidental binding which leads to activate APC/C. Estimating parameters such as chemical rates in the context of stochastic systems is challenging, as new methods and tools have to be used and developed, notably in statistical inference of Markov processes [19,33]. In our case, we use a Markovian approach to relate the chemical rates to the characteristics of the SAC observed at the cellular level. To guarantee an inhibition strong enough to prevent accidental binding, the probability that no activation occurred has to be high enough and plays a crucial role in determining the validity of the production rate parameters. In contrast to other quantitative studies of the SAC, we also study the premises of separation of sister chromatids during anaphase. As the SAC determines the amount of Cdc20 when anaphase starts, the time for activation of all APC/C located on kinetochores should not be neglected. Finally, in Figure 6, we obtain a range for the rates *λS *(which is the production rate at the beginning of SAC) and *k*_-1_. Actually, this range approximatively depends on the ratio $\rho =\frac{k_{-1}}{\lambda S}$, which satisfies

(36) 50≤ρ≤90.

The constraint *ρ *≥ 50 gives the minimum value required to produce enough Cdc20 molecules to activate the APC/C before 10 minutes. However, this ratio should not be too high, because an overproduction of Cdc20 could trigger a premature anaphase and thus *ρ *cannot also be too large, limited to 90. To close the domain Ω, a third constraint can be added by providing an upper bound for *k*_-1_. Because *k*_-1 _is given by the Smolu-chovski formula 2*πbD*[*MCC*], limiting the concentration [*MCC*] would precisely limit the chemical rate *k*_-1_. For example, when the number of MCC is in the range of 10000 (which corresponds to the Mad2 concentration of 200nM found in [34], and used in [16,17]), we obtain that *k*_-1 _≈ 24 and in that case, we approximatively get for the production rate

(37) 0.25≤λS≤0.5.

For example, fixing the value *λS *= 0.3, we find that anaphase is triggered after a mean time of 239 s (4 minutes), while the probability for no activation at time *t *= 20*min *is *P *= 0.96, which satisfies the biophysical constraints C1 and C2 described above. To close the domain Ω more tightly, several considerations can be suggested. The fact that once free/active, Cdc20 needs to find a securin-separase complex and bring it to the APC/C (alternatively find the APC/C first and not get ubiquitylated) might constrain the time somewhat further. We also did not take into account the effective time to get to equilibrium for Cdc20 or the time to clear inhibition of MCC. If the number of MCC is too large, this time cannot be neglected and would provide an explanation for overexpressing inhibitors that prevent anaphase [35]. It would be interesting to account for the dynamics of MCC [26,36], and how it can influence the transition phase between SAC and anaphase onset [2,35]. Finally, the present study can be extended to various cell geometry with different size and with different number of chromosomes.

## Authors' contributions

KDD and DH designed research and wrote the paper. KDD and DH performed research. All authors read and approved the final manuscript.
